# Prevalence and influencing factors of nonunion in patients with tibial fracture: systematic review and meta-analysis

**DOI:** 10.1186/s13018-020-01904-2

**Published:** 2020-09-03

**Authors:** Ruifeng Tian, Fang Zheng, Wei Zhao, Yuhui Zhang, Jinping Yuan, Bowen Zhang, Liangman Li

**Affiliations:** 1 Department of Orthopaedics, No.1 Hospital of China Medical University, Guangzhou, China; 2 Department of Orthopaedics, Shenyang Orthopaedic Hospital, Shenyang, China; 3 Department of Orthopaedics, No.4 Hospital of China Medical University, Guangzhou, China; 4 Department of Dermatology, No.1 Hospital of China Medical University, Guangzhou, China

**Keywords:** Tibia fracture, Nonunion, Prevalence, Influencing factors, Systematic review

## Abstract

**Objective:**

The aim of this study is to assess the prevalence of nonunion in patients with tibia fracture and the association between influencing factors and tibia fracture nonunion.

**Method:**

A database searches of PubMed, the Cochrane Library, EMBASE, China National Knowledge Infrastructure (CNKI), Weipu database, and Wanfang database from inception until June 2019 was conducted. The pooled prevalence, odds ratio (OR), and 95% confidence intervals (CI) were calculated with Stata software.

**Results:**

In this study, 111 studies involving 41,429 subjects were included. In the study of the relationship between influencing factors and tibia fracture nonunion, 15 factors significantly influenced the fracture union, including > 60 years old, male, tobacco smoker, body mass index > 40, diabetes, nonsteroidal anti-inflammatory drugs (NSAIDs) user, opioids user, fracture of middle and distal tibia, high-energy fracture, open fracture, Gustilo-Anderson grade IIIB or IIIC, Müller AO Classification of Fractures C, open reduction, fixation model, and infection.

**Conclusion:**

The prevalence of nonunion in patients with tibia fracture was 0.068 and 15 potential factors were associated with the prevalence. Closed reduction and minimally invasive percutaneous plate osteosynthesis (MIPPO) have the low risks of nonunion for the treatment of tibial fractures.

## Introduction

Fracture is a common disease that has a great impact on patients’ lives. Take Canada as an example, fractures and dislocations of the lower limb make up 38% of all injury admissions [1]. It is estimated that the disability from traffic accidents (the major cause of fractures) will rank the top three of all causes of disability by 2020 [2].

Fracture nonunion is one of the most common complications of fracture. The rate of fracture nonunion varies greatly in different anatomical locations of the fracture [3], with an average incidence rate of 4.93% [4]. Fracture nonunion is a chronic condition in terms of pain, and functional and psychosocial disability [5]. Nonunion of some fractures can reduce the quality of life and even increase the risk of death [3]. The cost of treatment for fracture nonunion was much more than that of fracture union [6, 7]. Other economic burdens caused by prolonged disability and downtime of job are more difficult to quantify but must be considered [8].

Good blood supply is an important condition for fracture union [1, 9]. Compared to other long bones with abundant blood vessels and soft tissue, the tibia with a longer subcu**t**aneous boundary normally has a poorer blood supply [10]. Therefore, tibial fracture has a higher risk of nonunion due to its special structure and blood supply. The definition of tibia fracture nonunion was no sign of union 9 months after surgical operation or no possibility of union if no further intervention was given assessed by surgeon [11].

Doctors need to know how to predict the risk of fracture nonunion and set up a plan to reduce the rate of fracture nonunion [8, 12]. In 2007, the “diamond concept” was introduced by Giannoudis et al., aiming to define what is required to achieve adequate fracture healing. This concept highlights the importance of three biological factors (osteogenic cells, osteoconductive scaffolds, growth factors) and a fourth factor known as mechanical stabilization. If one or more of these factors are altered, adequate fracture healing will be threatened [9, 13, 14].

Clinical and experimental studies have identified a number of potential factors that may help to predict fracture nonunion [15–18]. These factors include uncontrollable factors (for example, gender, age, underlying diseases, the way of injury) and controllable factors (for example, treatment method) [19, 20]. The uncontrollable factors of tibial nonunion may be similar to those of other anatomic sites. But there are too many influencing factors and even the same influencing factor may lead to different consequences in different anatomical positions [21]. For controllable influencing factors, the treatment of tibial fracture is also controversial [22]. Some doctors believe that intramedullary nailing (IMN) is the gold standard for the treatment of tibial fractures [23, 24]; however, most doctors consider that different treatment options have different advantages [25–28]. The use of non-steroidal anti-inflammatory drugs (NSAIDs) and the fixation of fibular fractures have also been considered as controversial factors for many years [29, 30].

Herein, we conducted a systematic review to explore the prevalence of nonunion in patients with tibia fracture and evaluate the association between influencing factors and tibia fracture nonunion. The study would provide valuable information for future prevention and treatment of tibia fracture nonunion.

## Methods

### Search strategy

The PubMed, Cochrane Library, EMBASE, CNKI (China National Knowledge Infrastructure), Wanfang database, and Weipu database were systematically searched, from inception to June 2019. The search keywords were “tibia” AND "fracture” AND “union OR nonunion OR disunion.” The manual search was performed through checking the reference lists of key studies and review articles to identify additional studies.

### Study selection

An overall literature search was performed and relevant studies were screened independently by two reviewers (Ruifeng Tian, Fang Zheng). Initially, all the titles and abstracts which were identified based on the keywords were screened. Secondly, full texts of articles which were selected from the first phase were reviewed. Finally, the articles which had contents suitable for data extraction were included in the systematic review. Disagreements between the two reviewers were resolved by a third reviewer (Wei Zhao) via discussion and consensus.

### Exclusion criteria

Exclusion criteria were as follows: neither English nor Chinese; animal model experiment; patients at the age of < 18; the cases of patients being lower than 10; insufficient information; duplicate publication; and obscure definition, such as delay union or mixed-descriptions of delay union and nonunion.

### Data extraction

Relevant data were extracted independently by two reviewers (Ruifeng Tian and Yuhui Zhang). Each of the following information was entered into a pre-designed form: first author’s name, publication year, basic information of patients (including history of medication, unhealthy habits and basic diseases), fracture type, operative information, the number of all tibia fracture patients, and the number of tibia fracture nonunion patients. The information of 19 potentially influencing factors were also exacted for comparison analyses, including age, gender, tobacco smoke, drink, body mass index (BMI), diabetes, nonsteroidal anti-inflammatory drugs (NSAIDs) user, opioids user, osteofascial compartment syndrome, fracture site, injury energy (low or high energy that causes tibia fracture), open fracture, Gustilo-Anderson grade, Müller AO Classification of Fractures (AO), debride time (the time from injury to debride), open reduction, fibula fixation, infection, and fixation models. Disagreements between the two reviewers were resolved by a third reviewer (Jinping Yuan) via discussion and consensus.

### Data analysis

Stata software (v12.0, Stata Corp, College Station, TX, USA) was used to assess all statistical analyses and a *p <* 0.05 was considered statistically significant. First, for exploring the prevalence of nonunion in patients with tibia fracture, the pooled prevalence and its 95% confidence intervals (CI) were calculated by using a random-effect model (*p* < 0.05, *I*^2^ > 50%), otherwise, or a fixed-effect model was selected (*p* > 0.05, *I*^2^ < 50%). When the prevalence rate in the included study was zero, double arcsine was used to deal with the data in case of data exclusion. Second, in the study of the association between potentially influencing factors and nonunion, the odds ratio (OR) and its 95% CI were calculated. To assess sources of heterogeneity, subgroup analyses were conducted, stratified by above 19 potentially influencing factors. Sensitivity analysis was performed by eliminating individual studies one by one. Publication biases were assessed by using the Begg’s test and Egger’s test.

## Results

### Characteristics of included studies

**Fig. 1:**
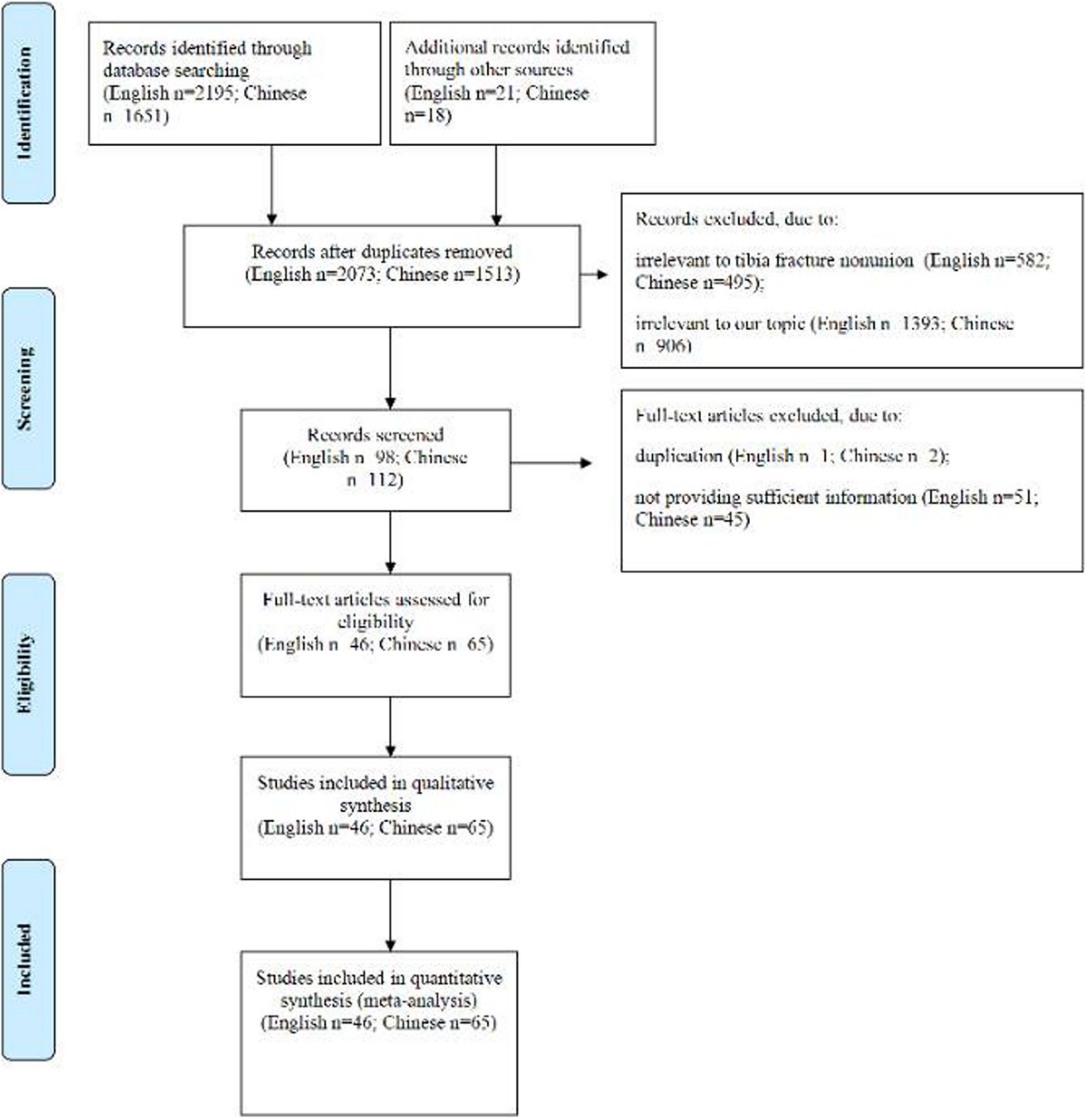
Flow diagram of the study selection process

A total of 3846 studies (2195 English and 1651 Chinese) were searched. Following selection process (Fig. 1), 111 studies were included in this systematic review and meta-analysis [6, 15, 16, 19, 31–136].

These studies were published between 1997 and 2019 from USA, China, Australia, Belarus, Canada, Egypt, France, India, Iran, Italy, Japan, Malaysia, Singapore, Turkey, and UK. There were 46 studies written in English and 65 studies in Chinese. The number of patients with tibia fracture ranged from 30 to 14638, and the prevalence of tibia fracture nonunion ranged from 0 to 42.7%. The basic information in all included studies were listed in Table 1.

**Table 1:** The basic information and prevalence of tibia fracture nonunion in each included study

Author	Year	Country	Age	Male	Female	Number of tibia fracture	Number of nonunion	Prevalence
Su CA [31]	2018	USA	40.4	225	102	284	19	0.067
Mehta D [32]	2018	USA	35.2	29	11	40	4	0.100
Milenkovic S [33]	2018	USA	43.5	20	12	32	6	0.188
Chang BS [34]	2018	China	23-57	38	26	60	7	0.117
Liu BQ [35]	2018	China	36.1	46	5	51	3	0.059
Zhang JS [36]	2018	China	49.4	60	34	94	5	0.053
Zhang QL [37]	2018	China	35	50	36	86	0	0.000
Yu JQ [38]	2018	China	42.4	65	39	94	5	0.053
Jin PF [39]	2018	China	57.6	90	107	197	26	0.132
Ge Y [40]	2018	China	39.3	50	42	92	2	0.022
Fang YS [41]	2018	China	45.2	49	13	62	1	0.016
Li J [42]	2018	China	35.5	46	39	70	2	0.029
Xu DY [43]	2018	China	40.9	38	26	64	3	0.047
Li ZT [44]	2018	China	52.4	48	42	90	1	0.011
Dailey HL [45]	2018	UK		739	264	1003	121	0.121
Singh A [46]	2018	Singapore	38.2	101	2	103	44	0.427
Galal S [47]	2018	Egypt	37.2	52	8	60	2	0.033
Javdan M[48]	2017	USA				231	12	0.052
Auston DA [49]	2017	USA	42	184	131	315	17	0.054
Zura R [50]	2017	USA	18-63	6273	6535	12808	944	0.074
Thakore RV [15]	2017	USA	36	364	102	486	56	0.115
Chan DS [51]	2017	USA	44	82	32	114	24	0.211
Xiong SR [52]	2017	China	42.5	82	66	148	8	0.054
Javdan M [48]	2017	Iran	35.9	45	4	49	3	0.061
BeytemürÔ [53]	2017	Turkey	40.6	52	21	73	1	0.014
Daolagupu AK [54]	2017	India	37.14	32	10	42	3	0.071
Garg S [55]	2017	India	38.9	5	31	36	4	0.111
Mukherjee S [56]	2017	India	40.3	26	14	40	3	0.075
Blair JA [57]	2016	USA	42.2	156	28	184	16	0.087
Burrus MT [16]	2016	USA		8132	6506	14,638	1758	0.120
Avilucea FR [58]	2016	USA	40.6	162	54	216	29	0.134
O'Halloran K [19]	2016	USA	39.3	93	289	382	56	0.147
Barcakë [59]	2016	USA				64	5	0.078
Shen J [60]	2016	China	45	54	71	125	0	0.000
Fang JH [61]	2016	China	36.8	40	16	56	2	0.036
Hao LS [62]	2016	China	19-67	67	15	82	2	0.024
Hu H [63]	2016	China	36.7	30	22	52	1	0.019
Liu JQ [64]	2016	China	43.2	44	16	60	1	0.017
Rao HR [65]	2016	China	35.7	35	15	50	2	0.040
Bai T [66]	2016	China	36.8	43	17	60	4	0.067
Zhao KP [67]	2016	China	35.6	41	17	58	1	0.017
Uchiyama Y [68]	2016	Japan	41.9	77	8	85	3	0.035
Gupta P [69]	2016	India	42.7	22	8	30	1	0.033
Piątkowski K [70]	2015	USA	49.5	24	17	45	12	0.267
Sun KF [71]	2015	China	43.1	32	20	115	7	0.061
Sun JQ [72]	2015	China	48	35	21	56	7	0.125
Ma N [73]	2015	China	45.4	334	246	580	82	0.141
Huang H [74]	2015	China	17-65	52	44	96	5	0.052
Huang PZ [75]	2015	China	32	43	13	56	1	0.018
Zhang YH [76]	2015	China	36.5	49	21	70	2	0.029
Luo BX [77]	2015	China	38.5	47	31	78	1	0.013
Wang B [78]	2015	China	41.2	39	33	72	2	0.028
Cui LH [79]	2015	China	37.5	53	21	74	2	0.027
Meng YH [80]	2015	China	31.6	19	35	54	1	0.019
Gong Y [81]	2015	China	16-39	38	32	70	11	0.157
Lian HK [82]	2015	China	35.1	51	43	94	4	0.043
Meena RC [83]	2015	India	37.5	32	12	44	2	0.045
Sathiyakumar V [84]	2014	USA	37.5	63	30	93	17	0.183
Li Y [85]	2014	China.	43.3	116	5	121	2	0.017
Dai QH [86]	2014	China	34.5	23	19	42	0	0.000
Wu ZH [87]	2014	China	48.5	32	18	50	1	0.020
Li ZZ [88]	2014	China	43.8	76	44	60	5	0.083
Ren Y [89]	2014	China	34.7	49	21	70	4	0.057
Luan HX [90]	2014	China	37.1	78	20	98	6	0.061
Zhang WJ [91]	2014	China	44	43	25	68	3	0.044
Heng WX [92]	2014	China	18-79	45	23	68	4	0.059
Yavuz U [93]	2014	Turkey	42	32	23	55	3	0.055
Lack WD [94]	2014	USA	45	92	71	163	13	0.080
Berlusconi M [95]	2014	Italy	45	42	18	60	5	0.083
Antonovaë [6]	2013	USA	52.5	378	475	853	99	0.116
Huang Q [96]	2013	China	36.9	80	40	120	3	0.025
Gong M [97]	2013	China	40.3	41	11	52	2	0.038
Lv YM [98]	2013	China	39.1	77	34	111	6	0.054
Xu YD [99]	2013	China	39	105	58	163	2	0.012
Clement ND [100]	2013	UK	77.9	63	170	233	23	0.099
Sitnik AA [101]	2013	Belarus	43	54	26	80	7	0.088
Yusof NM [102]	2013	Malaysia	24.5	52	6	58	10	0.172
Bishop JA [103]	2012	USA				32	1	0.031
Lin ZF [104]	2012	China	36.6	222	194	416	33	0.079
Zhang H [105]	2012	China	39.6	58	38	96	1	0.010
Jia QT [106]	2012	China	36	61	27	88	4	0.045
Zhou JL [107]	2012	China	53	43	9	52	10	0.192
Rouhani A [108]	2012	Iran	26.4	45	8	54	3	0.056
Vallier HA [109]	2011	USA	38.3	85	19	114	6	0.053
Zhu DK [110]	2011	China	18-76	53	31	84	3	0.036
Zhao DL [111]	2011	China	37.8	54	26	80	1	0.013
Liu F [112]	2011	China	32.6	32	14	46	4	0.087
Enninghorst N [113]	2011	Australia	42.4	66	23	89	26	0.292
Xu JQ [114]	2009	China	36.3	121	49	170	8	0.047
Li ZG [115]	2009	China	35.8	71	56	127	3	0.024
Mahmudi N [116]	2009	China	37	34	10	44	3	0.068
Deng HP [117]	2009	China	40.3	51	34	85	4	0.047
Dong JH [118]	2009	China	18-74	77	51	128	2	0.016
Fu KL [119]	2009	China				112	11	0.098
Zhou L [120]	2009	China	37.9	52	41	93	5	0.054
Lang ZY [121]	2009	China	33.6	51	16	67	2	0.030
Wu C [122]	2009	China	19-71	25	12	37	2	0.054
Li QM [123]	2009	China	37.6	168	51	219	6	0.027
Yokoyama K [124]	2008	Japan	34.6	70	14	84	17	0.202
Aderinto J [125]	2008	UK				54	3	0.056
Lu HY [126]	2007	China	34.5	158	98	256	9	0.035
Hu GZ [127]	2007	China	33.4	301	116	396	11	0.028
Zeng CJ [128]	2006	China	30.7	390	264	541	14	0.026
Zhang YL [129]	2006	China	35	73	25	98	9	0.092
Zhao XZ [130]	2006	China	43.8	52	26	78	5	0.064
Zhu GH [131]	2005	China	34	55	23	78	5	0.064
Harris I [132]	2005	Australia	34	124	39	163	13	0.080
Cole PA [133]	2004	USA				89	2	0.022
Bonnevialle P [134]	2003	France	40.8	34	15	49	8	0.163
Harvey EJ [135]	2002	Canada				110	13	0.118
Keating J [136]	1997	USA				112	9	0.080

### Pooled results, sensitive analysis, publication bias of the prevalence of tibia fracture nonunion

**Fig. 2:**
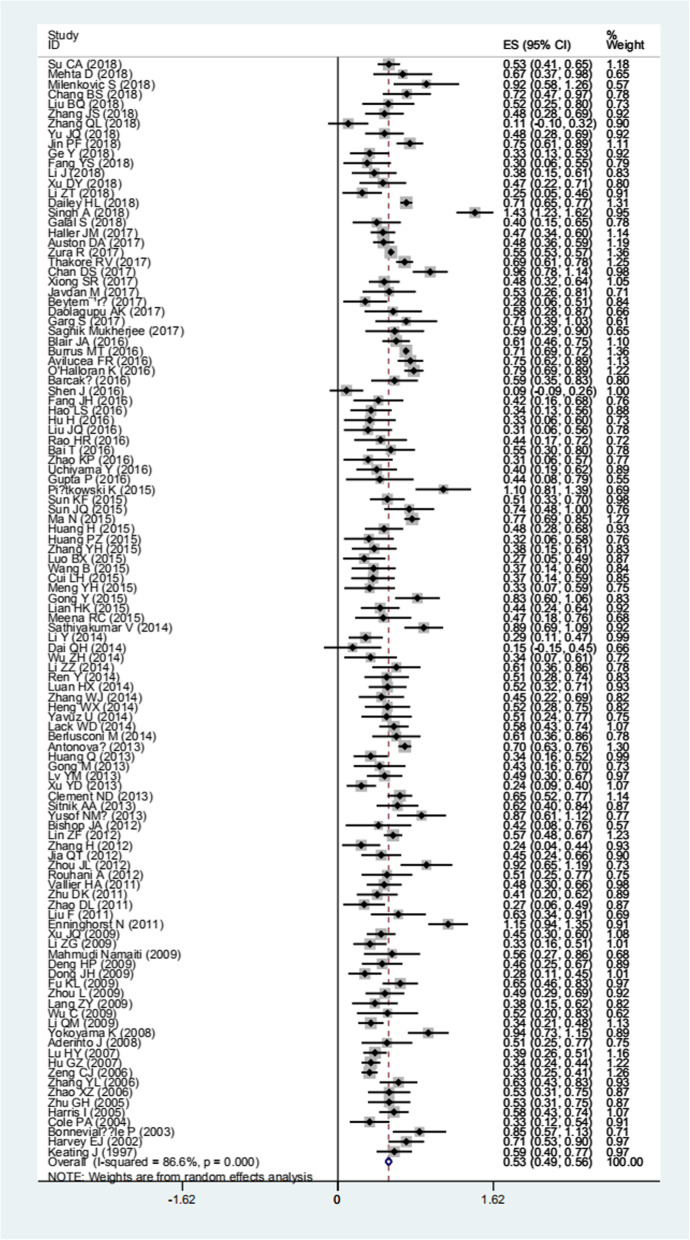
The forest plot of prevalence of tibia fracture nonunion

**Fig. 3:**
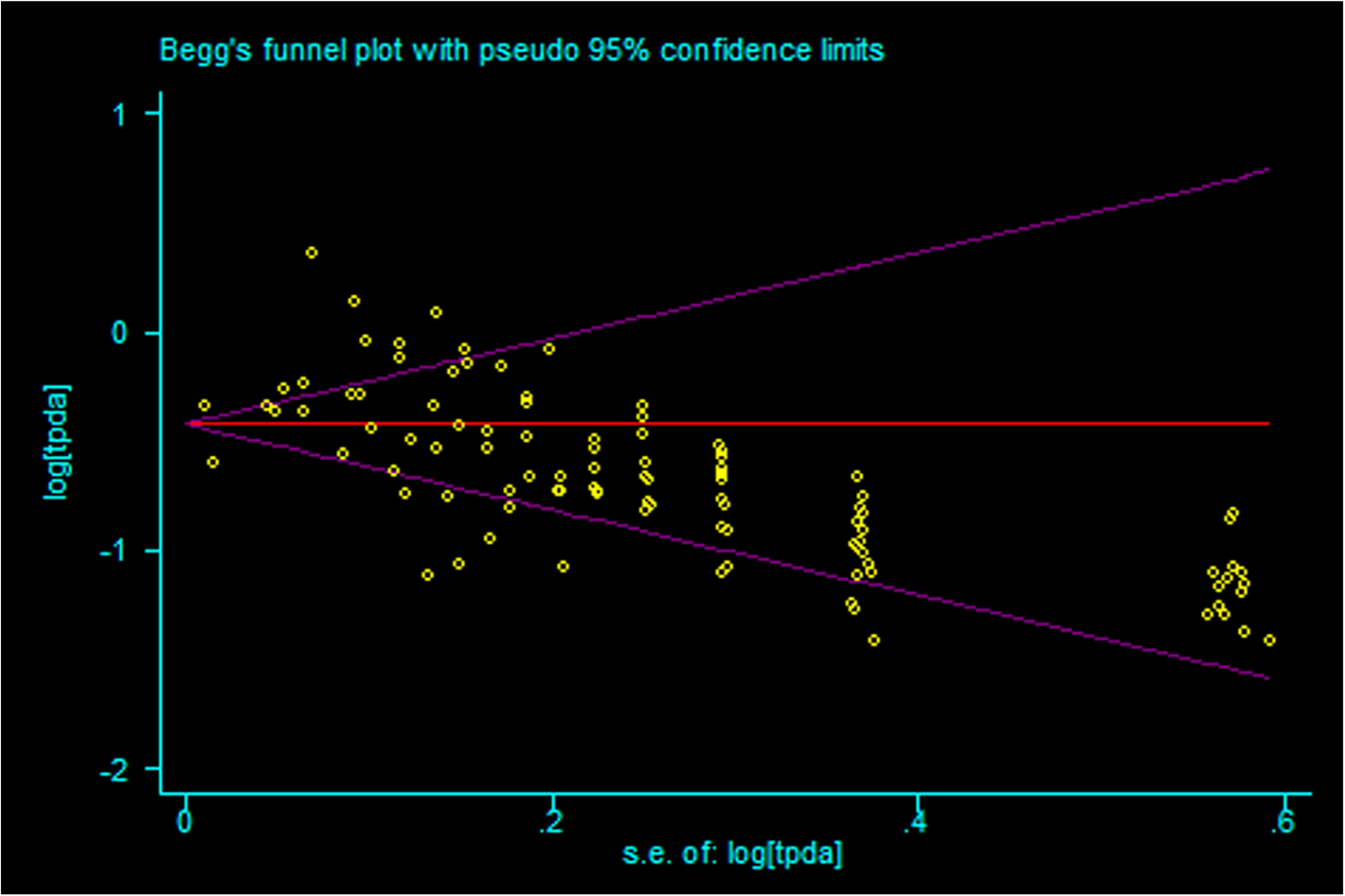
The publication bias of prevalence of tibia fracture nonunion

Based on the results of random-effects method (*p <* 0.05, *I*^2^ > 50%), the prevalence of nonunion from tibia fracture patient was 0.068 (95% CI 0.060–0.077) (Fig. 2, Table 2). The sensitive analysis demonstrated that there was no individual studies significantly affected the pooled results. The publication bias were found in pooled results (*t* = 3.19, *p* = 0.002) (Fig. 3).

**Table 2:** The pooled results and subgroup analysis of prevalence of nonunion from tibia fracture patient

		Number of study	*N*	*n*	Prevalence rate			Heterogeneity		Model
					effect size	lower limit	upper limit	*I*^2^	*p*	
Total		111	41429	3817	0.068	0.060	0.077	86.60%	< 0.01	Random
1. Age (year)	< 60	3	545	60	0.125	0.060	0.189	77.50%	0.012	Random
	> 60	3	316	65	0.204	0.160	0.249	0.00%	0.689	Fixed
2. Gender	Male	11	8186	790	0.131	0.104	0.159	77.80%	< 0.01	Random
	Female	11	8123	618	0.118	0.085	0.150	84.50%	< 0.01	Random
3. Tobacco smoker	Yes	8	2263	299	0.173	0.119	0.226	91.80%	< 0.01	Random
	No	8	12177	888	0.111	0.072	0.150	87.30%	< 0.01	Random
4. Drink	Yes	2	348	42	0.136	0.036	0.235	82.50%	0.017	Random
	No	2	12842	958	0.098	0.043	0.152	86.90%	0.006	Random
5. Body mass index	< 30	2	24466	2257	0.091	0.049	0.133	99.30%	< 0.01	Random
	> 30	2	3790	451	0.119	0.109	0.129	0.00%	0.557	Fixed
	30–40	2	2507	236	0.094	0.083	0.105	0.00%	0.441	Fixed
	< 40	2	26973	2493	0.091	0.053	0.128	99.20%	< 0.01	Random
	> 40	2	1283	215	0.160	0.020	0.218	87.80%	0.004	Random
6. Diabetes	Yes	4	347	73	0.221	0.178	0.267	8.50%	0.335	Fixed
	No	4	984	103	0.102	0.065	0.139	67.50%	0.046	Random
	Yes	3	371	58	0.153	0.116	0.189	0.00%	0.420	Fixed
	No	3	1197	144	0.117	0.099	0.135	59.90%	0.083	Random
8. Opioids user	Yes	3	1035	145	0.140	0.118	0.161	0.00%	0.694	Fixed
	No	3	522	58	0.097	0.031	0.164	78.40%	0.010	Random
9. Fracture site	Proximal	7	586	30	0.043	0.027	0.06	26.50%	0.254	Fixed
	Middle	7	724	115	0.146	0.080	0.211	84.60%	< 0.01	Random
	Distal	7	614	88	0.139	0.104	0.178	24.10%	0.253	Fixed
10. Injury energy	High	4	710	105	0.149	0.083	0.241	83.60%	< 0.01	Random
	Low	4	298	22	0.065	0.007	0.175	87.30%	< 0.01	Random
11.Open fracture	Yes	10	14037	916	0.062	0.049	0.074	56.20%	0.015	Random
	On	10	1985	390	0.197	0.145	0.294	84.80%	< 0.01	Random
12. Gustilo-Anderson grade^a^	I or II	9	680	57	0.070	0.051	0.089	31.30%	0.168	Fixed
	IIIA	9	394	55	0.130	0.097	0.163	0.00%	0.686	Fixed
	IIIB or IIIC	9	220	89	0.382	0.198	0.566	88.90%	< 0.01	random
13.Müller AO Classification of Fractures (AO) classification^b^	A	7	1039	69	0.059	0.027	0.090	68.90%	0.004	Random
	B	7	600	103	0.140	0.086	0.204	65.90%	0.007	Random
	C	7	285	54	0.158	0.078	0.260	74.50%	0.001	Random
14. Debride time	< 6 h	2	138	41	0.302	0.074	0.530	89.10%	0.002	Random
	> 6 h	2	49	20	0.405	0.268	0.541	0.00%	0.411	Fixed
15. Open reduction	Yes	9	573	48	0.075	0.043	0.107	52.40%	0.032	Random
	No	9	606	26	0.043	0.028	0.060	42.10%	0.086	Fixed
16. Fixation mode^c^	ORIF	41	6216	703	0.081	0.058	0.107	82.10%	< 0.01	Random
	IMN	51	12642	1326	0.054	0.040	0.070	77.30%	< 0.01	Random
	MIPPO	25	988	18	0.023	0.015	0.032	0.00%	0.835	Fixed
	External fixation		680	33	0.055	0.023	0.098	76.90%	< 0.01	Random
	Conservative treatment	4	116	22	0.134	0.003	0.409	92.10%	< 0.01	Random
17. Fibula fixed	Yes	7	166	11	0.073	0.027	0.140	53.20%	0.046	Random
	No	7	538	69	0.122	0.094	0.149	< 0.01	0.611	Fixed
18. Osteofascial compartment syndrome	Yes	3	210	31	0.134	0.088	0.179	61.90%	0.072	Fixed
	No	3	1359	162	0.105	0.058	0.151	85.40%	0.001	Random
19. Infection	Yes	2	217	84	0.510	0.155	0.866	93.80%	< 0.01	Random
	No	2	1366	119	0.076	0.022	0.129	92.80%	< 0.01	Random

^a^Gustilo-Anderson classification: grade I: clean wound < 1 cm in length; grade II: wound 1–10 cm in length without extensive soft-tissue damage, flaps or avulsions; grade III: extensive soft-tissue laceration (>10 cm) or tissue loss/damage or an open segmental fracture; grade IIIa: adequate periosteal coverage of the fracture bone despite the extensive soft-tissue laceration or damage; grade IIIb: extensive soft-tissue loss, periosteal stripping and bone damage, usually associated with massive contamination; grade IIIc: associated with an arterial injury requiring repair, irrespective of degree of soft-tissue injury

^b^AO classification of tibia fractures with designations of A: simple, B: wedge, C: complex

^c^*ORIF* open reduction and internal fixation, *IMN* intramedullary nailing, *MIPPO* minimally invasive plate osteosynthesis

### Subgroup analysis of prevalence of tibia fracture nonunion and comparison results

The prevalence of tibia fracture nonunion in different countries were of various (Tables 2, 3, and 4), for example, USA was 0.094 (95% CI 0.075–0.114), China was 0.047 (95% CI 0.039–0.057), etc.

**Table 3:** The comparison results stratified by 19 influencing factors

		Study	Comparison results				Heterogeneity		Model
			*p*	OR	lower limit	upper limit	*I*^2^	*p*	
1. Age (year)	> 60 vs. < 60	3	< 0.05	2.602	1.686	4.016	48.70%	0.142	Fixed
2. Gender	Male vs. Female	11	< 0.05	1.256	1.122	1.407	14.00%	0.311	Fixed
3. Tobacco smoker	Yes vs*.* No	8	< 0.05	1.692	1.458	1.964	49.30%	0.055	Fixed
4. Drink	Yes vs. No	2	0.083	1.367	0.960	1.947	0.00%	0.518	Fixed
5. Body mass index (BMI)	30 < BMI < 40 vs. BMI < 30	2	0.801	1.085	0.575	2.050	93.70%	< 0.05	Random
	BMI > 40 vs. BMI < 30	2	< 0.05	1.874	1.607	2.185	0.00%	0.660	Fixed
	BMI > 30 vs. BMI < 30	2	0.189	1.351	0.862	2.119	93.00%	< 0.05	Random
	BMI > 40 vs. 30 < BMI < 40	2	0.045	1.773	1.014	3.102	84.30%	0.012	Random
	BMI > 40 vs. BMI < 40	2	< 0.05	1.899	1.630	2.212	0.00%	0.892	Fixed
6. Diabetes	Yes vs. No	3	< 0.05	2.731	1.857	4.014	32.20%	0.229	Fixed
7. Nonsteroidal anti-inflammatory drugs user	Yes vs. No	3	0.018	1.536	1.076	2.194	0.00%	0.384	Fixed
8. Opioids user	Yes vs. No	3	0.012	2.010	1.166	3.468	0.00%	0.370	Fixed
9. Fracture site	Middle vs. Proximal	7	< 0.05	3.152	2.019	4.922	0.00%	0.788	Fixed
	Distal vs. Proximal	7	< 0.05	2.877	1.822	4.543	0.00%	0.911	Fixed
	Distal vs. Middle	7	0.670	0.932	0.673	1.290	0.00%	0.650	Fixed
10. Injury energy	High vs. Low	4	0.001	2.602	1.484	4.562	35.90%	0.182	Fixed
11. Open fracture	Yes vs. No	9	< 0.05	2.846	1.700	4.202	16.50%	0.296	Fixed
12. Gustilo-Anderson grade^a^	IIIA vs. I or II	9	0.005	1.831	1.204	2.784	0.00%	0.847	Fixed
	IIIB or IIIC *vs.* I or II	9	< 0.05	7.202	4.781	10.848	4.60%	0.394	Fixed
	IIIB or IIIC *vs.* IIIA	9	< 0.05	3.695	2.422	5.639	32.60%	0.168	Fixed
13. Müller AO Classification of Fractures (AO) classification^b^	B *vs.* A	7	0.010	2.522	1.249	5.930	54.20%	0.041	Random
	C *vs.* A	7	< 0.05	3.685	2.405	5.648	37.00%	0.160	Fixed
	C *vs.* B	7	< 0.05	3.569	2.428	5.325	39.60%	0.142	Fixed
14. Debride time	< 6 h vs. > 6 h	2	0.631	1.190	0.585	2.419	0.00%	0.520	Fixed
15. Open reduction	Yes vs. No	9	< 0.05	2.887	1.715	4.861	26.20%	0.220	Fixed
16. Fixation mode^c^	IMN vs. MIPPO	15	0.003	2.681	1.397	5.146	0.00%	0.980	Fixed
	IMN vs. ORIF	28	0.020	1.127	1.019	1.247	54.10%	<0.05	Random
	ORIF vs. MIPPO	7	0.010	3.495	1.351	9.045	0.00%	0.859	Fixed
	External vs. ORIF	10	0.115	0.506	0.217	1.182	54.00%	0.016	Random
	Conservative vs. ORIF	4	0.264	1.496	0.737	3.035	64.10%	0.062	Fixed
	External vs. IMN	10	0.993	1.006	0.266	3.806	55.40%	0.022	Random
17. Fibula fixed	Yes vs. No	7	0.435	1.317	0.659	2.634	47.60%	0.075	Random
18. Osteofascial compartment syndrome	Yes vs. No	3	0.106	1.420	0.968	2.173	80.30%	0.006	Fixed
19. Infection	Yes vs. No	2	< 0.05	11.877	7.461	18.906	52.10%	0.149	Fixed

^a^Gustilo-Anderson classification: grade I: clean wound < 1 cm in length; grade II: wound 1–10 cm in length without extensive soft-tissue damage, flaps or avulsions; grade III: extensive soft-tissue laceration (> 10 cm) or tissue loss/damage or an open segmental fracture; grade IIIa: adequate periosteal coverage of the fracture bone despite the extensive soft-tissue laceration or damage; grade IIIb: extensive soft-tissue loss, periosteal stripping and bone damage, usually associated with massive contamination; grade IIIc: associated with an arterial injury requiring repair, irrespective of degree of soft-tissue injury

^b^AO classification of tibia fractures with designations of A: simple, B: wedge, C: complex

^c^*ORIF* open reduction and internal fixation, *IMN* intramedullary nailing, *MIPPO* minimally invasive plate osteosynthesis

**Table 4:** Prevalence of nonunion from tibia fracture in different countries

	Number of study	*N*	*n*	Prevalence rate			Heterogeneity		Model
				Effect size	Lower limit	Upper limit	*I*^2^	*p*	
USA	19	30167	3083	0.094	0.075	0.114	93.40%	< 0.01	Random
China	68	7550	396	0.047	0.039	0.057	69.50%	< 0.01	Random
Australia	2	252	39	0.182	0.026	0.389	93.90%	< 0.01	Random
Belarus	1	80	7	0.088	–	–	–	–	–
Canada	1	110	13	0.118	–	–	–	–	–
Charlotte	1	163	13	0.08	–	–	–	–	–
Egypt	1	60	2	0.033	–	–	–	–	–
France	1	49	8	0.162	–	–	–	–	–
India	5	150	10	0.059	0.026	0.092	0	0.73	Fixed
Iran	3	152	9	0.059	0.022	0.097	0	0.99	Fixed
Italy	1	60	5	0.083	–	–	–	–	–
Japan	2	169	20	0.114	0.049	0.278	91.70%	0.001	Random
Malaysia	1	58	10	0.172	–	–	–	–	–
Singapore	1	103	44	0.427	–	–	–	–	–
Turkey	1	73	1	0.014	–	–	–	–	–
UK	4	1042	156	0.108	0.092	0.124	47.60%	0.126	Fixed

In the following comparisons of influencing factors (Table 3), each of the former prevalence of tibia fracture nonunion was significantly higher than the latter one (*p* < 0.05), i.e., > 60 years old (0.204) vs. < 60 years old (0.125), male (0.131) vs. female (0.118), tobacco smoker (0.173) vs. non-smoking (0.111), BMI > 40 (0.160) vs. BMI < 40 (0.091), diabetes (0.221) vs. no diabetes (0.102), NSAIDs user (0.153) vs. none NSAIDs user (0.117), opioids user (0.140) vs. none opioids user (0.097), fracture of middle segment (0.146) vs. proximal segment (0.043), fracture of distal segment (0.139) vs. proximal segment (0.043), high-energy injury (0.149) vs. low-energy injury (0.065), open fracture (0.197) vs. close fracture (0.062), Gustilo-Anderson grade I or II (0.070) vs. IIIA (0.130) vs. IIIB and IIIC (0.382), AO Classification A (0.059) vs. B (0.140) vs. C (0.158), open reduction (0.075) vs. close reduction (0.043), infection (0.510) vs. without infection (0.076). No significant difference was found between other comparisons (*p* > 0.05).

**Fig. 4:**
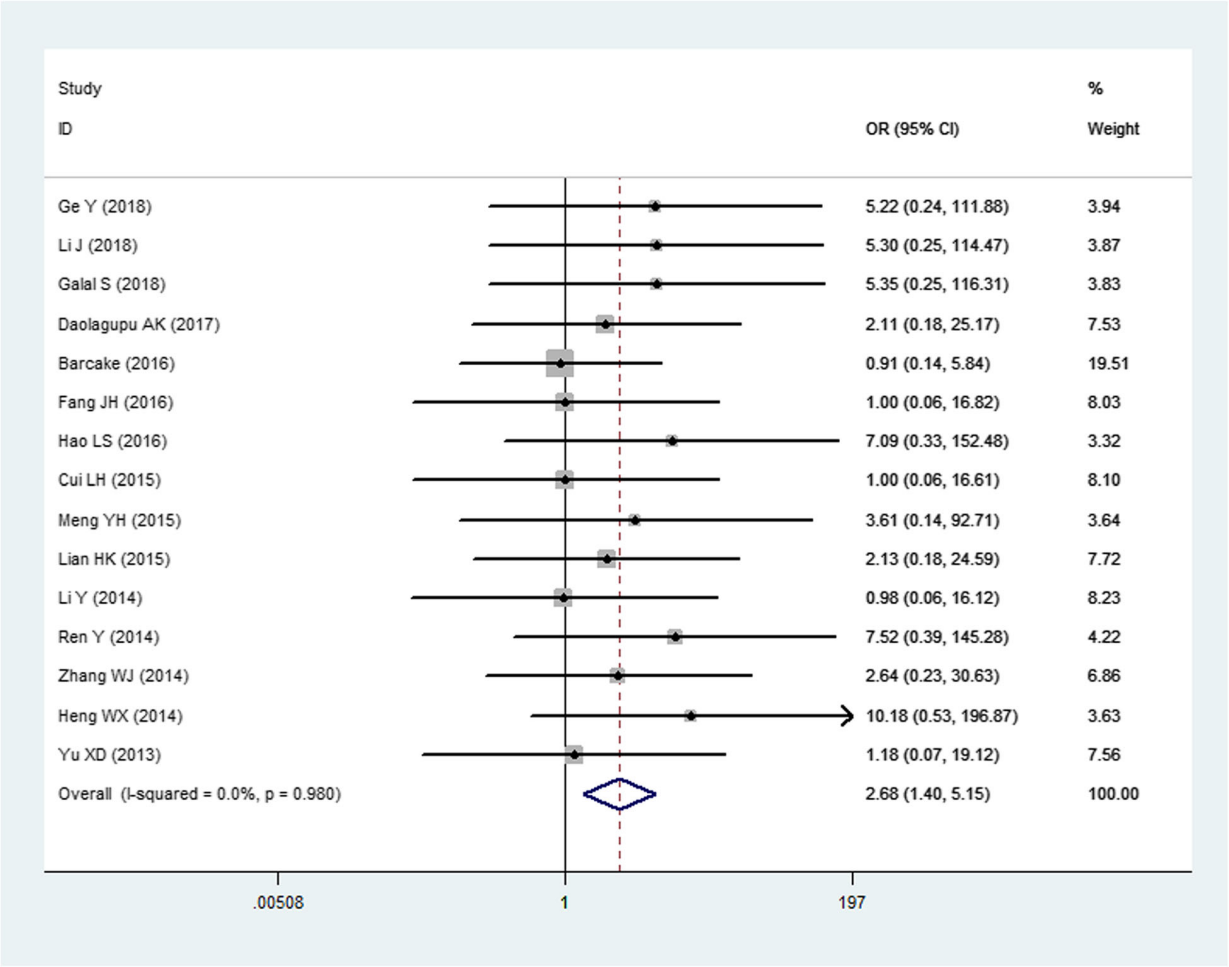
The comparison of MIPO with IMN

There were 5 fixation models of tibial fractures available, including open reduction and internal fixation (ORIF), intramedullary nailing (IMN), minimally invasive percutaneous plate osteosynthesis (MIPPO), external fixation, and conservative treatment. Significant difference was found between each other comparison of the following 3 fixation models, ORIF (0.081) vs. IMN (0.054) vs. MIPPO (0.023) (*p* < 0.05) (Fig. 4). No significant difference was found between external and ORIF, conservative and ORIF, or external and IMN (*p* > 0.05).

## Discussion

To our knowledge, this is the first systematic review and meta-analysis to estimate the prevalence of nonunion in patients with tibia fracture and the relationship between different influence factors and tibia fracture nonunion. The pooled prevalence of tibial fracture nonunion was 0.068. Different countries were in variety of prevalence, indicating a heredity disparity. The lowest prevalence was seen in Turkey (0.014) and next was Egypt (0.033); however, the numbers of included studies were so small that the conclusions were not so robust. There were 68 studies that were conducted in China involving 7550 tibia fracture patients and the prevalence of nonunion was 0.047. However, one study in Singapore, a country that has lots of Chinese population, presented a very high prevalence of tibia fracture nonunion 0.427, indicating other influencing factors other than heredity. In calendar year 2011, an inception cohort study in a large payer database of patients with fracture in the USA was conducted using patient-level health claims for medical and drug expenses compiled for approximately 12,808 patients, and the prevalence of tibial fracture nonunion was reported to be 0.074 [137]. In contrast, the present systematic review involved 30,167 patients in a total of 19 studies conducted in the USA and the prevalence was 0.094. The pooled results enabled a larger sample size and accessed more to the real conclusion.

Some influencing factors contributed to the nonunion of tibial fractures. In 2016, O'Halloran K et al. created a Nonunion Risk Determination Score (NURDS) to predict nonunion risk, based on 7 influencing factors (*p* < 0.05, OR > 2), including flaps, compartment syndrome, chronic condition(s), open fractures, male gender, grade of American Society of Anesthesiologists Physical Status, and percent cortical contact. While another 2 factors including spiral fractures and low-energy injuries can be predictive of union [19]. In our study, we found more influencing factors, including age > 60 years old, diabetes, opioids user, middle and distal fracture, high-energy injury, open fracture, Gustilo-Anderson grade IIIB and IIIC, and AO Classification C met above criteria (*p* < 0.05, OR > 2) and can be regarded as predictive indicators. Still, there were some other influencing factors, including male, tobacco smoker, BMI > 40, and NSAIDs user, partially predicated the risks (*p* < 0.05, OR < 2).

The present study showed that BMI > 40 and diabetes were the influencing factors of nonunion of tibia fractures. With the improvement of quality of life, the negative impact of obesity has gradually become a hot issue of concern. Obesity can lead to vitamin D deficiency, and whether there is a causal relationship between fracture nonunion and vitamin D deficiency is the focus of discussion [138, 139]. But we cannot ignore the fact that diabetes mellitus is closely related to obesity. In our study, the use of NSAIDs was also associated with fracture nonunion. Some experiments have proved that NSAIDs can temporarily inhibit the process of fracture union [140, 141]; however, other studies considered that the pain caused by fracture nonunion of patients led to their resorting to NSAIDs [142].

Our comparison showed that open reduction had a higher rate of fracture nonunion than closed reduction. In surgery, although open reduction can bring good fracture repair, but closed reduction can better protect blood supply and soft tissue. In addition, our study did not find a relationship between fibular fixation and nonunion rates of tibial fractures. However, Strauss EJ and Kumar A’ experiments on cadavers showed that fibular fixation can increase the stability of tibial fractures after surgery [143–145]. So whether it is necessary to fix the fibula for the treatment of tibial fracture accompanied by fibular fracture should be further determined.

The choice of fixation mode is a way to control the nonunion rate of tibial fracture artificially [146, 147]. We compared 5 fixation modes available. The nonunion rate of conservative treatment was the highest one compared with that of surgical treatment. This is obviously different from the lowest rate reported by Li H et al. [148]. This may be related to the insufficient number of articles in conservative treatment. Compared with traditional ORIF, IMN and MIPPO have lower fracture nonunion rate. No significant difference was found between external fixation and ORIF. Ebraheim NA et al. reported that IMN can achieve better healing effect in the treatment of tibial fractures, comparing to ORIF and external fixation [149]. MIPPO had the lowest nonunion rate of all fixation modes. It was proved that MIPPO can maximize the protection of soft tissue and bone marrow around the fracture site [150]. The above 5 fixation modes destroy the necessary conditions of fracture healing to varying degrees. However, it is worth mentioning that different options have different advantages in the treatment of tibial fractures [151, 152]. For example, in distal tibial fractures, more comminuted fractures would rather require open reduction than “simple” type A fractures. So it is unreasonable to only consider the nonunion rate of fracture of operation [148].

The systematic review and meta-analysis had made strict inclusion and exclusion criteria, but still had some limitations and bias which may be unavoidable. Firstly, due to different attentions of individual studies, the influencing factors were only extracted from partial studies with available data and some other influencing factors such as hemoglobin and bone defect were not mentioned. Secondly, different doctors and different hospitals had a variety of surgical technologies and conditions, which may cause unavoidable bias. Thirdly, the number of included studies and the data for meta-analysis were limited which may affect the final results to a certain degree. Fourthly, publication bias was found in the study. Therefore, the data from literature in other languages, more areas, and ongoing studies are required to reflect a more accurate and wide variation. Finally, non-randomized controlled trials (nRCTs) were involved in this systematic review. As a result, subjective factors may affect the result. More rigorous designs and large RCTs are required to make further verification.

In conclusion, the prevalence of nonunion in patients with tibia fracture was 0.068 and 15 potential factors were associated with the prevalence. Closed reduction and MIPPO have low risks of nonunion for the treatment of tibial fractures. A series of factors shed the light which may affect the union rate of tibial fracture for doctors’ reference, and provide the probability of nonunion of tibial fracture under different treatment schemes. The authors hope to help doctors assess the risk of nonunion and propose the most suitable treatment for patients with tibial fractures under different conditions.

## Data Availability

The datasets generated and analyzed during the current study are available from the corresponding author on reasonable request.

## Abbreviations

CNKI: China National Knowledge Infrastructure
OR: Odds ratio
CI: Confidence intervals
NSAIDs: Nonsteroidal anti-inflammatory drugs
AO: Müller AO Classification of Fractures
MIPPO: Minimally invasive percutaneous plate osteosynthesis
IMN: Intramedullary nailing
NRCTs: Non-randomized controlled trials
